# Targeting Focal Adhesion Kinase Using Inhibitors of Protein-Protein Interactions

**DOI:** 10.3390/cancers10090278

**Published:** 2018-08-21

**Authors:** Antoine Mousson, Emilie Sick, Philippe Carl, Denis Dujardin, Jan De Mey, Philippe Rondé

**Affiliations:** 1 Faculté de Pharmacie, Université de Strasbourg, 67401 Illkirch, France; antoine.mousson@etu.unistra.fr (A.M.); esick@unistra.fr (E.S.); philippe.carl@unistra.fr (P.C.); denis.dujardin@unistra.fr (D.D.); jan.de-mey@unistra.fr (J.D.M.); 2 CNRS UMR 7021, Laboratoire de Bioimagerie et Pathologies, 67401 Illkirch, France

**Keywords:** FAK, cancer signalling, PPI inhibitor, FAK inhibitor

## Abstract

Focal adhesion kinase (FAK) is a cytoplasmic non-receptor protein tyrosine kinase that is overexpressed and activated in many human cancers. FAK transmits signals to a wide range of targets through both kinase-dependant and independent mechanism thereby playing essential roles in cell survival, proliferation, migration and invasion. In the past years, small molecules that inhibit FAK kinase function have been developed and show reduced cancer progression and metastasis in several preclinical models. Clinical trials have been conducted and these molecules display limited adverse effect in patients. FAK contain multiple functional domains and thus exhibit both important scaffolding functions. In this review, we describe the major FAK interactions relevant in cancer signalling and discuss how such knowledge provide rational for the development of Protein-Protein Interactions (PPI) inhibitors.

## 1. Search for Inhibitors of Protein-Protein Interaction

### 1.1. Introduction

During the last two decades, considerable efforts have been made in the understanding of the molecular mechanisms of diseases. However, at present, only a small portion of proteins (around 400) involved in diseases have been explored as therapeutic targets [1]. Indeed, functional genomic studies have predicted that 3000–10,000 proteins are implicated in diseases [2]. Currently, therapeutic targets are primarily G protein-coupled receptors, enzymes, ion channels, transporters and nuclear receptors. These proteins are considered as easy to target because they have natural ligand-binding sites for endogenous agonists or substrate. It is therefore not surprising that pharmaceutical companies have extensively exploited these druggable proteins. However, the number of new drug approvals is stagnating at around 15–25 per year since five decades and the majority of launched drugs are derivatives of lead compounds with the same chemical signature [3]. Thus, the challenge in drug design and discovery is to identify new potential therapeutic targets in order to propose innovative therapies.

Among these potential new targets, protein-protein interactions (PPIs) are of considerable and growing interest. Indeed, PPIs are critical for many aspects of cell biology and are involved in physiological as well as pathological processes. However, targeting PPIs with small “drug-like” molecules is challenging for at least two main reasons. First, the contact surfaces involved in PPI are large (1500–3000 Å^2^) compared with those involved in small-molecule-protein interactions (300–1000 Å^2^) [4,5]. In PPI, affinity is mostly obtained by a multitude of often weak interactions. It is therefore difficult to design a small molecule that could bind tightly because of insufficient interactions. Second, the contact surfaces are usually flat and often lack the pockets that are characteristic of natural ligand-protein contact surfaces. Traditional ligand-binding sites are characterized by one or two large pockets with a typical volume close to 260 Å^3^ in contrast to PPI, which involve an average of six small pockets with a pocket volume around 54 Å^3^ [6]. It is therefore difficult to predict which pocket (s) at the protein-protein surface contact will be able to bind a small inhibitor, if any.

Despite these challenges, several evidences provide hope for discovering small drug-like molecules that would target PPI interfaces. Even though the protein-protein interfaces are large, mutational studies have shown that a small subset of residues involved is essential for high-affinity binding; they are called hot spots [7]. The disruption of the interactions mediated by these hot spots is efficient in inhibiting PPIs [8]. Hotspots often comprise side chains of tyrosine, tryptophan and arginine that all allow adaptive conformational changes to accommodate small molecules as well as strong hydrogen-bonding potential through nitrogen and oxygen atoms [9]. Traditional high-throughput screening (HTS), which is particularly suitable for the drug discovery in well-defined “druggable” pockets, was at present not very successful. This could be explained by the content of compounds used for screening that are derived mainly from chemistry efforts in pharmaceutical companies. These chemotypes are dominated by past drug-discovery research on GPCR, enzymes and traditional druggable targets. However, PPI interfaces strongly differ from druggable well defined binding pockets and therefore, the discovery of successful inhibitors requires that new chemotypes need to be included in HTS libraries in order to match to these novel class of target. Indeed, it is believed that except for close homologues, each protein-protein interface is different, so the chemotypes of their inhibitors are likely to be more isolated in chemical space. Computational modelling methods in combination with high-resolution X-ray crystal structures of protein complexes seriously improved the prediction of relevant small-molecule binding pockets on a protein-protein interface [10]. Additionally, advances in fragment-based lead discovery allow the rapid probing of the protein surface for small chemical fragments binding sites and therefore addresses the druggability issue [11]. It is possible that evaluating the structure activity relationship of compounds by nuclear magnetic resonance (NMR) as in fragment-based screening will be more successful than HTS when applied to PPI. Full ligand screening has already been used to find an inhibitor of Keap1/Nrf2 interaction able to initiate an anti-oxidant and anti-inflammatory response [12]. Furthermore, fragment-based methods have allowed discovering an inhibitor of STAT3 dimerization, which inhibits nuclear translocation of the protein and the transcription of pro-carcinogenic genes [13].

### 1.2. Successful Examples

Successful examples of PPI inhibitors have been reported in distinct biological pathways. One of those and probably the best-characterized are the inhibitors of the interaction between p53 and human protein double minute 2 (HDM2). MDM2 (the mouse homologue of HDM2), which is an ubiquitin E3 ligase was initially found to binds to the tumour suppression protein p53 leading to its proteasomal degradation [14]. p53 is inactivated by mutation or deletion of its gene in nearly 50% of human cancers. In the other 50% displaying wild-type p53, its tumour suppression function can be altered by several distinct mechanisms. One major inhibitory mechanism is mediated by the interaction between MDM2 and p53. This interaction has been mapped to the first 120 amino acid residues in the N-terminal domain of HDM2 and the 30 first amino acid residues in the N-terminal domain of p53. High-resolution crystal structure of MDM2 complexed with residues 15–29 of p53 was solved in 1996 [15] and reveals that p53/MDM2 interaction is mediated by a well-defined hydrophobic surface in MDM2 and three key hydrophobic residues in p53 (Phe19, Trp23 and Leu26) [15]. In search of inhibitors, the first class of potent and specific small MDM2 inhibitors were identified by HTS at Hoffmann-La Roche. These tetra-substituted imidazole inhibitors were named Nutlins. Nutlin-3, one of the most potent of these small molecules was reported to bind to MDM2 with IC50 = 90 nM and showed antitumour activity against xenografts in vivo [16]. Using NMR spectroscopy, Nutlin-3 was shown to insert its aromatic moieties into the same hotspot pockets in MDM2 where p53 binds [17]. Structure-activity relationship studies lead to the discovery of a refined Nutlin-3-derived compound, RG7112, which is the first MDM2 inhibitor advanced into clinical trials for the treatment of several human cancers [18]. RG7112 effectively binds to MDM2 with IC50 = 18 nM and inhibits the growth of cancer cell lines with wild-type p53 in vitro with IC50 = 0.18–2.2 μM. Moreover, it demonstrated good selectivity over cancer cell lines with p53 mutation (IC50 = 5.7–20.3 μM). Clinical evaluation of RG7112 after oral administration in phase I clinical trials have shown that the molecule is able to activate p53 signalling in human tumours leading to apoptosis [18]. These studies have provided a proof of concept that PPI inhibitors are efficient at the clinical level. However, several patients treated by RG7112 had severe adverse effects including neutropenia and thrombopenia. This haematological toxicity has prompted the development of a more selective compound, RG-7388 (Idasanutlin) actually in phase III of the clinical trial [19]. Several other MDM2 inhibitors are now making their way through clinical trials including AMG232 developed by Amgen (phase I/II) or MK-8242 developed by Merck (phase I) [20]. Their antitumoral activities are tested as single agents or in combination with traditional chemotherapeutic agents.

Another example of PPI inhibitors is small inhibitors of the Bcl-2 family in clinical trials especially for the treatment of patients with lymphoid malignancies. The Bcl-2 (B-cell lymphoma 2) family contains important regulators of apoptosis. These proteins govern mitochondrial outer-membrane permeabilization and form homodimers or heterodimers with other family members to mediate either a pro-apoptotic or a pro-survival signal. The effect of pro-apoptotic proteins Bak and Bax are blocked by the interaction with anti-apoptotic partners such as Bcl-2 and Bcl-X. This interaction is mediated by a hydrophobic BH3 domain in pro-apoptotic protein containing four key hydrophobic residues (Leu 59, Leu 63, Ile 66 and Leu 70) [21]. Disrupting this interaction by binding the anti-apoptotic Bcl-2 family proteins with small molecules BH3 mimetics induce apoptosis of cancer cells [22]. Abbott laboratories by using N heteronuclear single quantum coherence (HSQC) protein NMR, has developed Bcl-2 family inhibitors such as ABT-263 (Navitoclax). This compound binds Bcl-2 protein in vitro with subnanomolar affinities and inhibits tumour growth of chronic lymphocytic leukaemia (CLL) when combined with rituximab in phase II of clinical trial. However, navitoclax was found to induce thrombopenia by binding the anti-apoptotic Bcl-X protein with a similar affinity to Bcl-2 protein. Since the disclosure of navitoclax, several monoselective inhibitors of Bcl-2 has been developed such as venetoclax [23,24]. Venetoclax was shown to selectively bind Bcl-2 with high affinity and to induce apoptosis by Bax-Bak signalling. Its efficiency was demonstrated in a human xenograft model of B cell lymphoma and more recently in phase I/II of human clinical trial [24] as single agents or in combination with traditional chemotherapeutic agents. This new class of Bcl-2 inhibitor reduces thrombocytopenia risk associated with the inhibition of Blc-X while conferring a rapid reduction of tumour in CLL patients.

Thus, PPI inhibitors may represent an interesting alternative to target protein kinases. Indeed, the majority of reported protein kinase inhibitors are ATP-binding competitors which bind to the active kinase conformation (type I inhibitors) resulting in often limited kinase selectivity due to the high degree of conservation within the ATP-site. Searching for competitive inhibitors rather than ATP-competitive inhibitors may be a more rational approach. At present, more than 1500 ATP-dependent enzymes have been identified with 420 Ser/Thr kinases and 90 Tyr Kinases. Moreover, the high intracellular ATP concentrations necessitate high intracellular concentrations (μM) of the inhibitor to effectively inhibit the target, even when the affinity of the inhibitor is in the nanomolar range [25]. On the other hand, PPI inhibitors frequently have subnanomolar affinity to the target and their efficiency has already been proven at this concentration in many cases. Thus, selectivity of these inhibitors may be better than kinase inhibitors that compete with ATP in the substrate-binding site. Moreover, inhibition of kinase activity does not alter protein scaffold properties and subsequent signalling pathways. PPI can be targeted with small molecules that would have a pharmacokinetic profile that is different from the current known PPI inhibitors. Currently, the majority of small molecules in clinical trials are orally available drugs despite of their high molecule weight and hydrophobic clusters. However, PPI inhibitors design is challenging and requires a perfect understanding of the structure activity relationship.

## 2. FAK Structure and Interaction

**Figure 1:**
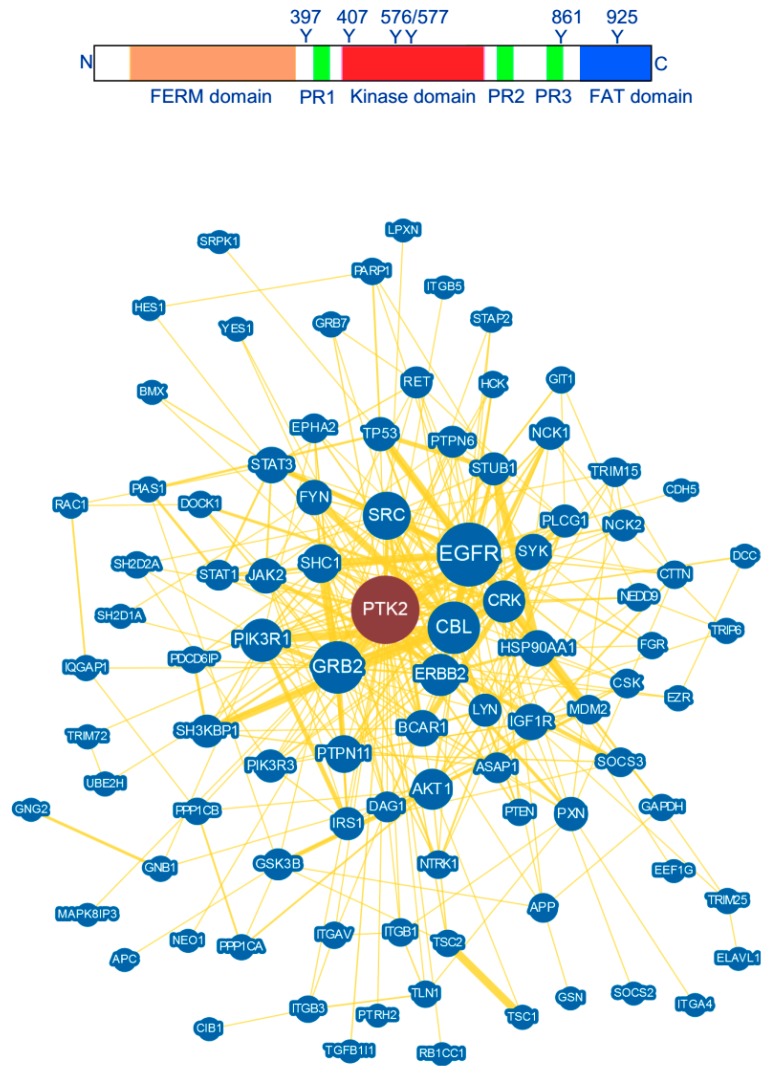
The main structure domains of FAK. Important sites of tyrosine phosphorylation are also indicated. Graphical network of FAK protein interactions identified by BioGRID based on a compilation of publications referring to protein and genetic interactions. Circles with layers closest to the centre are more highly connected.

More than twenty years ago, FAK was first described as a substrate of the oncogene product of the sarcoma virus (v-Src) and as a protein highly phosphorylated upon clustering of Integrins at focal adhesions [26,27,28]. Soon after, the group of William Cance at the University of North Carolina found an increase in FAK mRNA in invasive and metastatic human tissues [29]. Subsequently, analysis of FAK protein expression in a variety of human tumours revealed increased levels of FAK that correlated with the invasive potential of these tumours [30]. This lead to intensive research for the mechanisms of FAK activation and the signalling pathways regulated by the kinase. FAK is a ubiquitously expressed non-receptor cytoplasmic tyrosine kinase composed of an N-terminal FERM (band 4.1, ezrin, radixin, moesin homology) domain, a central kinase domain, several proline-rich domains and a C-terminal focal adhesion targeting (FAT) domain (Figure 1). FAK is primary implicated in the regulation of signals initiated at sites of Integrin mediated cell adhesion to the extra-cellular matrix as well as signals triggered upon growth factor receptors activation [31]. The first experimental evidence implicating FAK in tumour formation and progression was obtained by using conditional knock-out mice with selective *fak* deletion in the epidermis [32]. This proof of concept experiment led to the development of strategies aimed at inhibiting FAK with the hope to reduce tumour progression. Thus, the development of FAK antagonists, as anti-cancer therapy, led to several small inhibitors of FAK kinase function that are currently undergoing clinical trials.

Nevertheless, besides its kinase function, FAK possess also scaffolding functions that are highly relevant in cancer signalling [33]. Indeed, according to the Biological General Repository for Interaction Datasets (BioGRID) [34,35], FAK is involved in none less than 235 interactions. Nevertheless, some of these interactions are redundant because they were characterized via different methods and by different laboratories. For example, Paxillin both interacts with the FAT domain of FAK and is a substrate for its kinase activity. Thus, the total number of unique FAK interactions identified until now is rather 125 (Figure 1). The BioGRID data base considers as an interaction any direct physical binding of two proteins, co-existence in a stable complex and genetic interaction. Therefore, the term interaction does not necessary involve a physical interaction between two proteins as these interactions are recorded using various techniques including affinity capture-MS, affinity capture-Western, biochemical activity, co-fractionation, co-purification, FRET or two-hybrid. For example, the affinity capture method identifies an interaction when a protein is affinity captured from cell extracts by an antibody and the associated partner identified either by mass spectroscopy or by Western blot. Thus, for FAK, some interactions were identified by the two-hybrid system while many others were characterized by the affinity capture-Western method and therefore may also be indirect as part of a signalling complex. Interactions identified by high-throughput two-hybrid systems need to be further characterized in order to establish their biological effect on a defined system and thus will not be fully addressed in this study. In this review, we will rather focus on direct FAK interactions with a particular interest for those involved in cancer initiation and progression. These interactions and their consequences on FAK activation and signalling will be described in details and we will examine how the knowledge of the structural motifs involved in these interactions could be the basis for development of PPI inhibitors.

## 3. FAK Structural Determinant for the Search of Potent FAK Inhibitors

### 3.1. Major Interactions at the FERM Domain

#### 3.1.1. FAK Interaction with Growth Factor Receptors and Mechanism of FAK Activation

**Figure 2:**
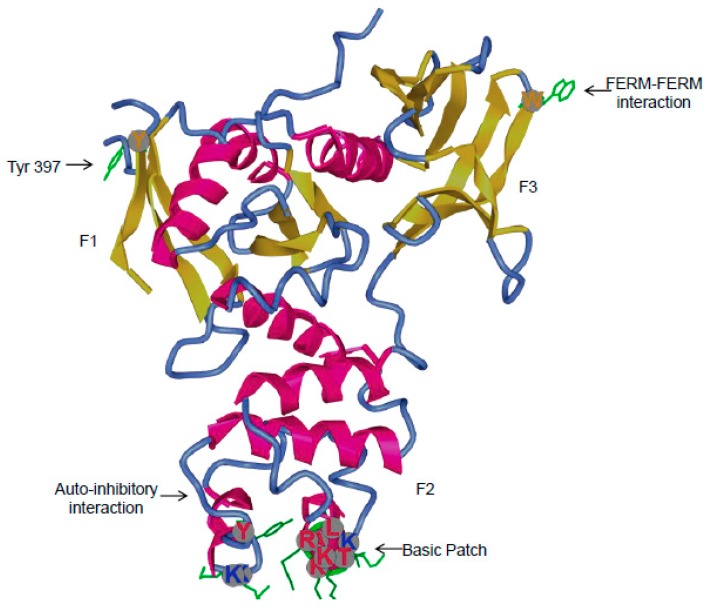
Structure of the FERM domain of FAK from the protein databank (accession code 2AL6). The FERM domain displays three lobes, F1, F2 and F3. The structure includes the Tyr397 auto-phosphorylation site which is located between the FERM and kinase domain. In the F2 lobe, residues belonging to basic patches important for FAK activation are highlighted and their side chains coloured in green. The auto-inhibitory interaction implicates residues from the F2 lobe of the FERM domain and C lobe from the kinase domain. The Trp266 implicated in FERM-FERM interaction necessary for FAK dimerization is also highlighted. Proteins associating with the FERM domain are shown in the table.

The best characterized mechanism that promotes FAK activation involves Integrin receptor clustering upon cell binding to the extracellular matrix which has been shown to involve binding of the β Integrin cytoplasmic domain to FAK [27,36,37]. Further analysis of Integrin-FAK interactions revealed that the cytoplasmic tail of the β1 Integrin directly stimulates FAK activity in vitro, this activity being increased after deletion of the FERM domain of FAK suggesting a mechanism of FAK autoinhibition [38]. Recently, the β4 Integrin-FAK interaction was mapped to 11-amino-acid region ahead of the FAK Tyr397 site [39]. FERM domains usually promote the coupling of cytoskeletal structures to the plasma membrane. In the case of FAK, recent studies have shown that the regulation of FAK activity entails an intramolecular association of the FERM domain with the kinase domain, which then blocks the accessibility of the Tyr397, the autophosphorylation site. Indeed, the crystal structure of a FAK fragment containing the FERM domain and the kinase domain in its auto-inhibited form reveals that this interaction requires the F2 lobe of the FERM domain notably the residues Y180 and M183 and the c-lobe of the kinase domain centred on F596 [40]. This mechanism of action implies that lipid-protein and/or protein-protein interactions in the FERM domain are necessary to release the FERM-kinase interaction (Figure 2). Thus, many components able to induce FERM-kinase domain opening have been proposed which include Phosphatidylinositol-4,5-bisphosphate (PtdIns (4,5) P_2_ [41] the tetraspanin TM4SF5 [42] and growth factor receptors. Among the latter both PDGFR, EGFR, IGF-1R, c-Met and RET were shown to form a complex with the FERM domain of FAK [31,43,44,45], thus suggesting that the resulting increases in FAK activation, may be due to the relief of the FERM-kinase auto-inhibition. In the case of RET kinase, a transactivation mechanism has been highlighted which involves reciprocal phosphorylation of FAK Tyr575/576 by RET and RET Tyr905/1062 by FAK. Recently, HER2 was shown to directly interact with the FAK-FERM-F1 lobe thereby promoting phosphorylation of FAK Tyr 397 [46]. On the other hand, c-Met promotes FAK phosphorylation at Tyr194 leading to FAK activation. Constitutive activation of FAK by replacement of Tyr194 with the phosphomimetic E194 can be overcome by mutations of the basic patch KAKTLRK of the F2 lobe of the FERM domain, indicating that the interactions between the phosphorylated Tyr194 and these basic residues may allow FAK activation through relief of its autoinhibition [47]. Consequently, the addition of HGF stimulates invasion of MDCK cells through Matrigel. VEGF also stimulates FAK activation leading to the association of the cytoplasmic tail of VE cadherin with the FAK FERM domain thus enabling β-catenin phosphorylation to promote junctional disassembly and vascular permeability. As the VEGF dependency of FAK activation is lost upon Y180A/M183A mutation it is believed that in endothelial cells, VEGF activates FAK in a conformation-dependent manner [48]. Early experiments have also demonstrated a role of the FAK-Ezrin interaction in the control of FAK activity as in kidney-derived epithelial cells, ezrin promoted FAK phosphorylation at Tyr-397. Mapping experiments showed that FAK amino acids 1–376 were required for optimal Ezrin binding [49].

Using a FRET-based biosensor for monitoring FAK activation it was also confirmed that PIP2 triggers a change in FAK conformation state which likely represents unbinding of the FERM-kinase domain [50]. However, PIP2 has been shown to be necessary for FAK clustering at the membrane and partial opening of FAK conformation without complete release of auto-inhibitory interactions [51]. Thus, it remains possible that increased PIP2 levels simply induce clustering of FAK molecules necessary for the FAK trans-phosphorylation. In this case, other mechanisms such as FAK dimerization mediated by traction force or FERM-FERM interactions would be necessary to release the closed FERM-kinase conformation state. Force-mediated FAK activation has been shown to involve a basic patch in the FERM domain able to prevent FAK inhibition induced by an interaction between FERM domain acidic sites and Myosin II [63]. Moreover, FAK activation by dimerization which has been first described in 2002 [64] require an intermolecular FERM-FERM interaction around residue W266 stabilized by the FAT (Focal Adhesion Targeting) binding to a basic patch K_216_AKTLRK located in the FERM F2 lobe [65]. The FERM domain is also involved in direct FAK binding to Integrin-containing endosomes thereby regulating adhesion induced FAK activation and cancer related processes such as anchorage-independent growth [66]. This report established an unprecedented FAK activation pathway distinct from the signalling role of Integrin/FAK complexes at FAs. Nevertheless, the relationship between FAK and the endocytic and membrane trafficking processes was first described by Gundersen’s group, who found an interaction between FAK and dynamin at FAs during forced FA disassembly [52]. Latter it was shown that dynamin is specifically recruited at FAs by a direct interaction with the FERM domain of FAK thus allowing dynamin phosphorylation at Tyr 231 by Src [67].

Other mechanisms of FAK autoinhibition have also been unveiled. First, based on early structural data on the FERM domain together with different linker segments, the Tyr397 site was shown to be inaccessible because of an interaction with the F1 lobe of the FERM domain and inhibition of Src binding due to sequestration of the SH3 binding site via an intramolecular interaction with the F3 lobe [68]. Second, it was recently reported that relief of FERM autoinhibition can be provided by changes in pHi which mediated deprotonation of the H58 residue of the FERM domain and subsequent conformational changes that modulating the accessibility of Tyr397 [69]. In the autoinhibited conformation, it is possible that the FAK FERM domain may also bind to the actin nucleating protein Arp3 and promotes the recruitment of the Arp2/3 complex to nascent adhesions in a kinase-independent manner [70]. As FAK is needed for nascent adhesion assembly, this complex could be implicated in the transition of nascent adhesion into FA. This interaction implies FAK K38 a residue previously implicated in FAK activation [53] suggesting that FAK binding to Arp2–3 may be one element for the release of FAK auto-inhibition. This FAK/Arp3 interaction has been recently shown to be involved in haptotaxis [54].

#### 3.1.2. FAK Control of Cell Polarity and Migration

Several mechanisms seem to be implicated in the control of wound-induced cell polarity. Indeed, it was first described that FAK directly interact and phosphorylate the actin regulatory protein N-WASP thereby promoting cell migration [55]. Integrin-mediated FAK phosphorylation at Tyr397 creates a binding site for p120RasGAP which allows FAK to subsequently phosphorylate p190A thus creating a molecular complex that regulate Golgi orientation and cell polarization [71]. Latter, other experiments indicated that another complex composed of FAK, RACK1 and PDE4D5 located at nascent adhesion is also involved in Golgi orientation and direction sensing. The interaction of FAK with RACK implicates residues 139–140 located in the FERM domain and mutation of these residues lead to impaired nascent adhesion formation, Golgi reorientation, polarization and chemotactic invasion in squamous cell carcinoma [56]. Moreover, a peptide disrupting RACK1-PDE4D5 binding mimics the effect on Golgi re-orientation. This FAK/RACK1/PDE4D5 complex likely signals to the guanine nucleotide exchange factor EPAC, which in turn activates its small GTPase target Rap1. Interestingly, this signalling complex has been shown to mediate cell invasion in BRAF-mutated melanoma suggesting a link between polarity efficiency and cell invasiveness [72]. The transcription factor Nanog also mediate cell invasion via direct binding to the N-terminal domain of FAK with subsequent increased formation of filopodia/lamellipodia [58]. Finally, FAK is also able to activate ETK, a member of the Bruton’s tyrosine kinase (Btk), via interaction of the F1 sub-domain of FERM and the PH domain of ETK. This interaction is involved in the regulation of Integrin-mediated endothelial and metastatic carcinoma cell migration [57].

#### 3.1.3. FAK Functions in the Nucleus

The FERM domain of FAK contain different elements such as nuclear localization signals (NLS) and nuclear export signals (NES) that regulate the shuttling of FAK between the nucleus and FA. In the F2 lobe, beside the ^216^KAKTLRK^222^ basic patch which mediates interaction with other proteins, another partially overlapping surface exposed basic patch involving K190/191, K216/218, R221 and K222 has been shown to mediate FAK localisation to the nucleus. This raised the intriguing possibility that in the absence of FAK activation signal involving binding of c-Met, PIP2 or other molecules to these basic residues, FAK will be mainly located to the nucleus due to unmasking of the NLS signal. Besides, another basic patch namely R177/178 located in the F2 lobe is also consistent with NLS signal activity [59]. Interestingly, FAK can be post-translationally modified by addition of a small ubiquitin-related modifier (SUMO) at the Lys152 residue via PIAS1 (Protein Inhibitor of Activated STAT1) binding to the FERM domain [60]. Sumoylation is often associated with nuclear import and sumoylated FAK present in the nuclear fraction is associated with increased FAK activity.

Normal cells require adhesion to the ECM in order to survive and grow a process that is dependent on FAK activation. Indeed, many reports have shown that inhibition of FAK leads to the onset of apoptosis [73,74]. Besides, FAK is over-expressed in many cancer cells [75] and a hallmark of the tumourigenesis process is the resistance to anoikis a form of apoptosis induced by the loss of contact to the ECM. Survival signals can be transduced either by the activation of the PI-3K/AKT pathway, cell cycle progression through cyclin D1 or inhibition of apoptotic pathways such as caspase and FADD-dependent pathways or p53 signalization. Receptor-Interaction Protein (RIP) is a serine/threonine kinase that contains a death domain and associates with the death receptor complex to provide apoptotic signals. This signal could be inhibited via direct interaction of the N-terminal domain of FAK with the death domain of RIP [61]. Moreover, early studies demonstrated that FAK transduces survival signals from the ECM via inhibition of p53 as a consequence of direct interaction between the N-terminal domain of FAK and the N-terminal domain of p53 [62]. Later, it was shown that FAK-meditated survival requires the F2 lobe of the FERM domain for nuclear localization of FAK, the F1 lobe for binding to p53 and the F3 lobe for interactions with Mdm2 [59]. This mechanism implies ubiquitination of p53 and Mdm2-mediated proteasomal degradation thus keeping p53 at low levels to facilitate cell survival. At this time, this was one of the first kinase-independent roles of FAK described which requires only the scaffolding function of FAK.

#### 3.1.4. Inhibitor of FERM Interactions

The search for inhibitors of PPIs can be made by selectively targeting mechanisms that modulate the kinase activity or specific interactions with known regulators of cancer-related pathways. For the first purpose, one should target protein-protein interactions that play a role in the regulation of kinase activity in order to achieve targeting specificity. In this aim, a protein pharmacophore model for the FAK-related kinase Pyk2 F3 FERM domain was recently generated and served as a template for the screening of the LeadQuest database. This led to the identification of a small compound that directly bind to the Pyk2 FERM domain and inhibited the Pyk2-stimulated glioma cell migration [76]. Nevertheless, because the survival pathway is critical for cancer progression inhibiting FAK/p53 interactions using PPIs could therefore represent an efficient alternative of classical kinase inhibitors. The search of PPI inhibitors to reduce FAK activity has been pioneered by the group of William Cance at the Roswell Park Cancer Institute in Buffalo. They first described a sequence of 7-amino-acid residues in the N-terminal proline-rich domain of human p53 necessary for FAK binding and show that a peptide containing these residues together with a TAT sequence for cell penetration was able to reduce viability of breast and human colon carcinoma cell lines [77]. Having validated this interaction as a potential therapeutic target, they performed computer modelling of the p53 peptide containing the 7 amino acid sequence, docked it into the three-dimensional structure of the FERM domain of FAK involved in the interaction with p53 and screened small molecules from different libraries for docking into the FAK-p53 pocket [78]. Several small compounds were selected and notably the compound 1-benzyl-15,3,5,7-tetraazatricyclo [3.3.1.1~3,7~] decane, named Roslin2 were able to reactivate p53 in colon cancer cells both in vitro and in-vivo and to sensitize cancer cells to doxorubicin and 5-fluorouracil.

Another successful example came from the same group targeting the FAK-Mdm-2 interaction. Mdm-2 is a p53 target involved in p53 proteasomal degradation which was found to bind FAK via the F3 lobe of the FERM domain [59]. Using the structures of the FAK FERM domain and Mdm-2, macromolecular techniques were used to model the FAK-FERM interaction which serves as a template for the virtual screening of 200,000 small molecule compounds from NCI database. This strategy helped them to identify a 5’-O-Tritylthymidine compound called M13 that significantly decrease viability in different cancer cells [79]. Several other protein-binding sites have been identified in the FERM domain. For example, pharmacological inhibition of FAK-mediated FAK localisation in the nucleus promotes anti-inflammatory properties via an interaction between the FERM domain of FAK and GATA4 thus preventing cytokine-stimulated VCAM-1 transcription [80]. Moreover, in addition to the known effect of Nanog, a homeobox transcription factor, on FAK transcription, Nanog also directly bind the N-terminal domain of FAK leading to Nanog phosphorylation which in term lead to altered cancer cell filopodia and lamellipodia formation [58].

The interaction between FAK and IGF-1R was also partially characterized. It appears that this interaction encompasses FAK amino-acid 127–243 and the N-terminal part of IGF-1R [81]. Using the strategy described above, modelling and targeting the FAK-IGF-1R interaction led to the identification the lead compound INT2-31 [81]. This compound was shown to effectively disrupt FAK/IGF-1R interaction in melanoma leading to reduce cell viability and proliferation and to the induction of cell arrest and apoptosis. Importantly, in-vivo in tumour xenografts, the compound effectively decreased AKT signalling, resulting in significant melanoma tumour regression [82].

### 3.2. Major Interactions at the Kinase Domain

#### 3.2.1. Role of Tyr397 in FAK Catalytic Activity and Interactions with Binding Partners

**Figure 3:**
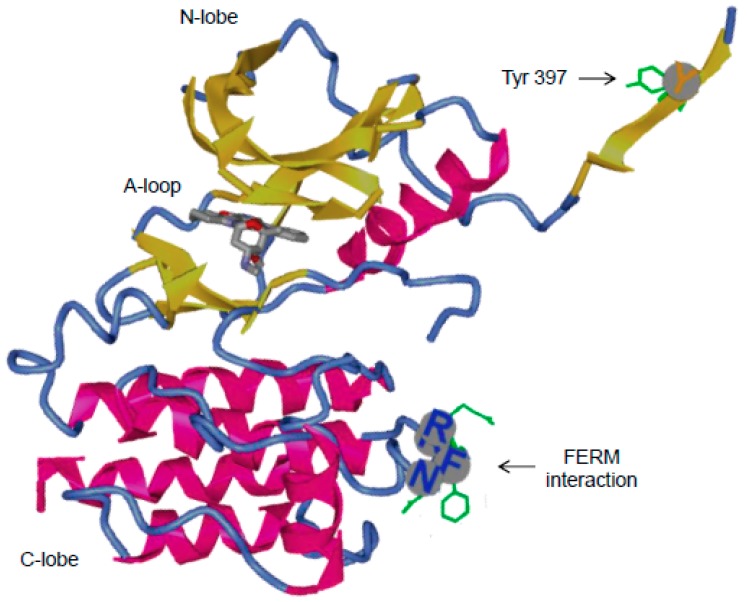
Structure of the kinase domain of FAK from the protein databank (accession code 2JOJ). The kinase domain displays the C lobe, N lobe and the activation loop. In the C lobe, residues important for FAK auto-inhibition that interact with the F2 lobe of the FERM domain are highlighted and their side chains coloured in green. Proteins associating with P-Tyr397 and the kinase domain are shown in the table.

Tyr397, which is located between the FERM and the kinase domain, is the major FAK activation site (Figure 3). FAK phosphorylation at the Tyr397 residue occurs via both intra and intermolecular processes [64,83]. Early studies have shown that mutation of Tyr397 to Phe altered cell adhesion and leads to reduced focal adhesion turn-over and cell motility in many cell types [84,85]. Phosphorylation of FAK tyr397 creates a motif that is recognize by Src family kinases thereby inducing several downstream signalling pathways [86]. Depending on the cell type, Src family kinases phosphorylate other tyrosine residues namely Tyr407, Tyr576, Tyr577 in the kinase domain, Tyr 861 in the junction between the kinase and FAT domain and Tyr925 in the FAT domain [87,88,89]. Tyr407 negatively regulates FAK activity whereas Tyr567 and 577 are necessary for full catalytic activity of the kinase function [87,90]. In addition to autophosphorylation, FAK is implicated in the phosphorylation of several other FA associated proteins including Paxillin [91] and p130CAS [92] although it is not entirely clear whether it is the kinase function of FAK or Src kinase in complex with FAK which phosphorylates the downstream substrates. Paxillin and p130CAS has both been implicated in the regulation of migration and invasion pathways. Indeed, cell motility is dependent on the ability of FAK/Paxillin to control the fine spatiotemporal regulation of FA turn-over [93]. Thus, overexpressing Paxillin that is mutated at FAK phosphorylation sites inhibits the turnover of focal contacts and cell motility. On the other hand, the p130CAS/Rac1 pathway has been rather implicated in lamellipodia and invadopodia formation and thus in mechanisms underlying cell invasion [94].

FAK phosphorylation at Tyr397 and FAK activation is also important in tumour-associated endothelial cells where it regulates endothelial cell motility, angiogenesis, endothelial and cell sprouting [48,106,107,108]. Thus, besides promoting phosphorylation of FA proteins, FAK can be also localized at adherent junctions in endothelial cells where, upon VEGF activation, FAK phosphorylate directly β catenin at Y142 [48] and indirectly, via Src activation, VE cadherin at Y658 [109]. This has important implications for the metastatic process as this lead to disruption of β-catenin-VEcadherin interactions thereby altering endothelial barrier function and increasing vascular permeability. Localised increases in vascular permeability constitute preferential discrete sites for cancer cell homing due in part to endothelial FAK-mediated expression of E-selectin [110].

The majority of FAK binding partners at the kinase domain involved the binding of SH2-containing proteins to FAK phosphorylated at Tyr397 which includes Src [95] but also PI3K [96], phospholipase Cγ (PLCγ) [97], growth-factor-receptor-bound protein-7 (GRB7) [98], p120 RasGAP [71] and the suppressor of cytokine signalling, (SOCS) [101]. Upon binding to phosphorylated FAK at Tyr397, increased PLCγ1 enzymatic activity was observed [97] which may be needed for calcium-dependent FA disassembly [111,112], while Grb7 binding induced also cell motility, a process that require FAK-mediated Grb7 phosphorylation [102]. FAK promote also cancer progression by activating the oncogenic Ras pathway. Negative regulators of Ras, include p120RasGAP, a GTPase activating protein, which associates directly with Ras and promotes hydrolysis of the bound GTP, thereby inhibiting Ras activity. In malignant astrocytoma, it was reported that overexpression of FAK promote FAK association with p120RasGAP thereby promoting Ras activity through a competitive inhibition [103]. This association involves Tyr397 as shown by mutation analysis. FAK association with p120RasGAP also facilitates FAK-mediated phosphorylation of p190RhoGAP that regulates polarity in migrating cells [71]. Autophosphorylation of FAK at Tyr 397 may also provide binding sites for proteins that negatively regulate FAK activity. Indeed, SOCS proteins presumably bind FAK to Tyr397 and both the Src homology 2 (SH2) domain and the kinase inhibitory region domains of the SOCS proteins contribute to SOCS binding to FAK [101]. This led to the inhibition of FAK kinase activity and to the polyubiquitination and degradation of FAK. Another example of negative regulation of FAK is represented by the binding of FIP200 (FAK family interacting protein of 200 kDa) to the kinase domain of FAK which regulate cell cycle progression [105]. Finally, the Phosphatase and Tensin Homolog (PTEN) tumour suppressor interact with FAK in a FAK Tyr397 dependent manner and reduces the phosphorylation at this site thereby reducing cell migration and invasion processes [99,100].

#### 3.2.2. FAK Inhibitors: Targeting the ATP Binding Site

FAK phosphorylation and activation drives many cancer-related processes. Therefore, based on this knowledge, two strategies can be developed to inhibit FAK function in cancer cells. The first is to inhibit the kinase function of FAK and the second to alter Src binding to FAK because this event is at the basis of the full FAK kinase activity. Pharmacological companies like Novartis or Pfizer have developed several kinase inhibitors of FAK which are almost all small ATP-competitive molecules. One of the first compounds developed was TAE-226 which exhibit nanomolar inhibitory activity toward FAK but inhibit also insulin-like growth factor-I receptor kinase [113,114]. In vitro, this compound prevented cell invasion through Matrigel, reduced cell proliferation, increased apoptosis and interestingly, it also enhanced docetaxel-mediated growth inhibition [115]. In vivo, the therapeutic efficacy of TAE-226 was related to induction of apoptosis of tumour-associated endothelial cells and reduction of tumour cell proliferation and microvessel density. The crystal structure of the kinase domain of FAK in complex with this molecule revealed that the DFG motif adopts a helical conformation which is stabilized by interactions with TAE-226 [116]. Nevertheless, despite substantial selectivity due to this conformation, the development of this molecule was later abandoned due to off-target effects. PND-1186 is another FAK inhibitor that targets the ATP-binding site but displays also inhibition against FLT-3 and ACK1 [117]. This compound, now named VS-4718, is on phase I clinical trials alone for patient with metastatic cancer or in combination with paclitaxel and Gemcitabine for patient with pancreatic cancer. At the same time, Pfizer developed PF-271 a compound that displays nanomolar inhibitory activity toward FAK and Pyk-2 but show high selectivity against a broad range of other kinases [118]. In multiple human xenograft models this compound display dose dependent antitumor efficacy. It should be noted that in pancreatic ductal adenocarcinoma, PF-271 inhibit cell migration in tumour cells but had no effect on cell proliferation at doses 10 times higher than those required to inhibit FAK catalytic activity and migration/invasion processes [119]. Remarkably, in a phase I clinical trial study, PF-271 was reported to be safe and well tolerated up to 125 mg twice-per-day and show significant stabilisation of tumour progression in some patients [120]. On the other hand, PF-228 another highly selective FAK inhibitor developed by Pfizer, inhibited both chemotactic and haptotactic cell migration in many cell lines but did not inhibit cell proliferation or induce apoptosis at similar concentrations [121]. GlaxoSmithKline developed also a FAK inhibitor named GSK2256098 that, in early studies, displayed minor clinical responses in mesothelioma subjects. However, based on recent work indicating that anti-cancer therapy using selective BRAF inhibitors may activate FAK pathway [122,123], GSK2256098 was also tested in combination with the MEK inhibitor trametinib in a Phase Ib study in subjects with advanced solid tumours and in combination with the frizzled class receptor (SMO) inhibitor vismodegib in a phase II study in patients with progressive meningiomas harbouring mutations in SMO or NF2. Although the second study is still on-going the first one was ended because it did not demonstrate improved efficacy in subjects treated with the combination of GSK2256098 plus trametinib compared to that observed with GSK2256098 monotherapy Finally, the late generation kinase inhibitor PF-04554878, now named VS-6063, was reported to stabilize some patients with colorectal or ovarian tumours in phase I clinical trial and is actually tested in the phase I/Ia study in combination with the anti-PD-1 Pembrolizumab for patients with advanced solid malignancies, in a phase I/Ib study in combination with placlitaxel for patients with advanced ovarian cancer and in two phase II studies, one for patients with KRAS mutant non-small cell lung cancer and one for subjects with malignant pleural mesothelioma based on their Merlin status. Moreover, with the idea of fighting cancer therapeutic resistance, a triple combination of VS-6063, carboplatin and paclitaxel is currently tested in a phase I/II clinical trial named ROCKIF (Re-sensitization of Carboplatin-resistant Ovarian Cancer with Kinase Inhibition of FAK) for the treatment of patients with ovarian cancer. Taken together, these results suggest that targeting the FAK kinase activity with new generation FAK inhibitors may be promising drug especially when added in combinatorial therapies. Although the selectivity is a well-known problem of many competitive inhibitors of the ATP binding site, in the case of FAK inhibiting the closely related kinase Pyk2 may be useful as lessons from FAK^−/−^ cells have shown increased Pyk2 activity due to compensatory effect. However, several reports suggest that although FAK kinase activity is necessary for cell motility, it may be not essential for cell proliferation and survival. Indeed, as already mentioned, these later effects are most certainly mediated by the FERM domain of FAK through regulation of p53 and the Receptor-Interaction Protein.

#### 3.2.3. Inhibiting FAK P-Tyr Binding to SH2-Containing Protein

To avoid selectivity problems typically found with competitive inhibitors of the ATP binding site, inhibiting the scaffolding function of FAK with the use of PPI inhibitors can be an attractive alternative approach. Specific interactions between SH2 domain-containing proteins and their phosphotyrosine counterparts play a significant role in tyrosine kinase signalling pathways. Thus, blocking the interaction between SH2 domain and its binding protein may inhibit overactive signalling pathways in cancer cells. For example, using structure-based virtual screening of the National Cancer Institute chemical libraries, small inhibitors of Stat3, targeting the Stat3 SH2 domain bound to a Stat3 phosphotyrosine peptide, were discovered [124]. In the case of FAK, with a similar strategy to the one used to characterize Roslin2 and M13, Vita Golubovskaya and her colleagues at Buffalo used computer modelling together with in-silico screening to target the FAK Y397 site and identify two compounds, Y11 and Y15 which display high inhibition of FAK autophosphorylation. As described previously Y397, upon phosphorylation, is involve in FAK interactions with Src, PI3K and some other SH2-containing proteins. Therefore, it is expected that such compounds would alter apoptosis and migration/invasion pathways. Indeed *in-vitro*, Y11 dose-dependently decreased viability, adhesion and clonogenicity in colon and breast cancer cell lines while significantly reducing tumour growth in a colon cancer cell mouse xenograft model [125]. Moreover, in in-vivo models of both colon cancer and glioblastoma, Y15 display synergy with 5-FU and temozolomide chemotherapy respectively [126,127]. Finally, in early preclinical studies investigating the pharmacokinetic profile, Y15 did not display toxic effect at doses effective to promote anti- tumoural results. Therefore, altogether, although the selectivity of these compounds has to be clearly evaluated, the use PPI inhibitors to target FAK-dependent pathways seem quite promising.

### 3.3. Major Interactions at the Prolin-Rich Domains

#### 3.3.1. Role of the FAK PR Domain in the Invasion Pathway

FAK contains three prolin-rich (PR) domains mediating many protein interactions (Table 1): PR1 located within the N-terminal domain between the FERM and the kinase domain surrounding Pro371/374; PR2 and 3 located both into the C-terminal domain between the kinase and the FAT domain and surrounding Pro712/715/718 and Pro875/878/881, respectively. PR1 domain is involved in the interaction with Trio a GEF protein called Trio that regulates FAK activity, focal adhesion dynamics and the cytoskeleton organization [128]. The PR2 domain is involved essentially in the Src/p130Cas-mediated migration and invasion processes. Thus, both Tyr397 and the PR2 domain of FAK are necessary for Src targeting and activation at FAs [129]. It should be also noted that the same pattern of interaction has been observed for PI3K where, although many data indicate interactions between its SH2 domain with FAK phosphorylated at Tyr397, the SH3 domain of the p85 units is also interacting with the PR domain of FAK [130]. Proteins of the CAS (Crk-associated substrate) family act essentially as scaffolds to regulate the formation of protein complexes controlling different processes such as migration, differentiation, cell cycle and apoptosis. p130Cas has been shown to be a component of the Integrin signalling machinery involved in cell motility. The role of FAK in the targeting and phosphorylation of p130Cas has been a matter of controversy. Indeed, the SH3 domain of p130Cas has been shown to be either essential [131] or not [132] to the targeting of p130Cas to FAs. Because p130Cas exists at FAs in a macromolecular complex with FAK and Src, both of which sharing PR domain and tyrosine kinase activity, the relative role of FAK and Src in addressing and phosphorylating p130Cas are difficult to assess. One model postulates that FAK autophosphorylation at Tyr397 allows the recruitment of Src via its SH2 domain, while interaction of the SH3 domain of p130Cas with the PR2 domain of FAK is needed for FAK-mediated p130Cas localisation at FA and subsequent phosphorylation by Src. Nevertheless, the “Cas-family C-terminal homology” (CCH) domain may adopt a tertiary structure similar to the FAT domain of FAK and recently it was demonstrated that this CCH domain have the ability to function as a FA targeting domain [133]. Another candidate for p130Cas targeting to FAs is the LIM protein Ajuba, which associates with the CCH domain of p130Cas and may localize p130Cas to nascent adhesive sites in migrating cells [134]. Therefore, from these data, it appears that p130Cas localize to FA via both FAK dependant and independent mechanisms, although the main one at least in FAK-expressing cells, seems to be linked to the interaction of the SH3 domain of p130CAS with the PR2 of FAK. The activation of p130Cas is linked to both Rac-mediated lamellipodia formation and subsequent increase migration and to transduction of ECM stiffness into intracellular stiffness leading to an increase in cyclin D1 expression thereby modulating cell cycling [135]. In v-Src-transformed cells, this pathway has also linked MMP expression and cell invasion [94]. In these cells, FAK was also shown to form a complex with Calpain and p42ERK thus promoting FAK proteolysis and FA turn-over. This interaction is mediated by the PR2 domain of FAK and is necessary for efficient targeting of Calpain to FAs [136].

Cortactin is another important protein that directly interacts with both FAK PR2 and PR3 domains thereby being phosphorylated at its tyrosine sites Tyr 421 and Tyr 466 which leads to FA dynamic regulation [147]. Cortactin is a major signalling component of the specialized matrix-degrading organelles termed invadopodia. Despite its role in the signalling pathway regulating invasion, FAK is generally not located at invadopodia [137,148,149] although it has been described in inavdopodia from MCF10A-CA1 breast cancer cells [138]. Nevertheless, FAK may promote invasion via focalised matrix degradation at FAs, a process that involves a complex comprising FAK, p130CAS and MT1-MMP. This complex is formed by a direct interaction between FAK and MT1-MMP implicating both PR1 and PR2 domains [139]. In addition, FAK interact via its PR3 domain with Endophilin A1, a protein implicated in endocytosis. This interaction promotes Src-dependent phosphorylation of Endophilin A1 leading to inhibition of Endophilin/Dynamin interactions thereby reducing endocytosis of MT1-MMP which contributes to ECM degradation [140].

The shuttling of FAK in and away from FAs is crucial for FAK regulation of the migration/invasion pathway driven in part by the spatial-temporal control of Src activity. In cancer cells, inhibition of FAK leads to altered Src trafficking via at least two major pathways. The first implies a relocation of Src from FAs to invadopodia through its interaction with the FAK homologue Pyk2 leading to enhanced matrix degradation [148]. The second pathway is engaged to maintain cell viability when the FAK/Src pathway is severely compromised and involves c-Cbl-mediated autophagic targeting of Src [141]. The targeting of Src to autophagosomes is controlled by the autophagy regulator Ambra1 (Activating Molecule in Beclin1-Regulated Autophagy) and requires Ambra-binding proteins such as Dynactin 1 and IFITM3 (Interferon-Induced Transmembrane protein 3) [142]. Nevertheless, in the presence of FAK, Ambra bind to the FAK PR3 domain thereby controlling the spatial regulation of FAK/Src at FAs. Indeed, a FAK P875A/P881A mutant with disturbed Ambra binding properties increases the time-residency of FAK/Src at FAs leading to enhanced adhesion.

#### 3.3.2. FAK as a Regulator of the Rho-GTPase Family

Other SH3-containing domains that interact with FAK involve critical regulators of the Rho-GTPase family, which are important molecular switches that regulate cell motility. GTPase activating proteins (GAP) and guanine-nucleotide exchange factors (GEF) bind to these domains to regulate Rho, Rac or Cdc42. For example, the Rho regulator, implicated in cytoskeleton reorganisation, termed PSGAP (Pleckstrin homology and the SH3 domain containing rhoGAP) has been shown to also interact with FAK. This protein binds to a sequence located near Pro 859 in Pyk2 close to the FAT domain and presumably to the equivalent PR3 sequence in the FAK structure, although this has not been formally demonstrated [150]. The Graf protein (GTPase Regulator Associated with FAK) also binds via its SH3 domain to the PR3 site to regulated actin organisation, a mechanism required for FAK function in promoting haptotaxis motility [104]. The Arf GAP proteins are another family of regulators that stimulate GTP hydrolysis bound to ADP-ribosylation factors (Arfs) thereby controlling membrane trafficking and cytoskeletal organization. ASAP1, an Arf GAP protein containing SH3, ANK repeat and PH domains localized at peripheral adhesions has been shown to contain an SH3 domain that binds to FAK and translocates from FAs to dorsal ruffles to regulate the Actin cytoskeleton [143]. Finally, the intracellular scaffold protein containing both IQ and GTPase-activating protein 1 motifs (IQGAP1) was recently found to interact with FAK in MDCK cells under conditions promoting RhoA activation thereby controlling FAs assembly [144]. Is not known whether this interaction is direct or not but one possibility could be a direct interaction between the WW motifs of IQGAP1 and the PR motifs of FAK.

On the other hand, FAK associates with the guanine nucleotide exchange factor PDZ-RhoGEF to modulate Rho/Rho kinase II signalling necessary for focal adhesion dynamic at the tail and trailing edge retraction but the structural determinants of FAK required for this interaction were not described [145]. Both PDZ-RhoGEF and LARG, a GEF containing a PH domain, can be phosphorylated by FAK in response to thrombin, thereby enhancing the activation of Rho [151]. The cycle of activation/inactivation of Rho family GTPases depend on the coordination of GEFs and GAPs. As discussed here FAK is associated with the recruitment at FA and their phosphorylation of several GEFs necessary for full RhoA activation but also with GAP proteins like Graf or PSGAP necessary for RhoA inactivation. It is unknown why FAK via different motifs such as Tyr397, PR domain or FAT domains interact with different GEF or GAP. However, it is very unlikely that FAK bind to these molecules at the same time. It seems rather that FAK controls the spatial-temporal activation of GEFs and GAPs to modulate cell contractility during the migration process.

#### 3.3.3. Role of FAK Ser732

The effects induced by the phosphorylation of FAK at Ser 732, that is close to PR2 have been addressed in several cell types. The first reports describe that FAK phosphorylation at this site is important for microtubule organization and microtubule-dependant mechanisms such as nuclear translocation, mitosis and neural cell migration [146,152]. Latter, CDK5-dependant FAK phosphorylation at Ser732 specifically localized at the spindle microtubule was shown to be implicated in cancer cell proliferation and migration. Of note this process appears independent of Integrin engagement [153]. Besides, CDK5, the Rho-dependent kinase ROCK also directly phosphorylates FAK downstream of VEGF signalling [154]. Finally, an interaction between Dynein, Paxillin and FAK reported in both mouse endothelial and NRK cells was shown to involve FAK phosphorylation at Ser 732 [155].

#### 3.3.4. Inhibiting the FAK PR Domain Binding to SH3-Containing Protein

At the moment, no PPI inhibitors targeting FAK interactions via its PR domains has been developed. However, this strategy may be valuable as protein domains that bind proline-rich motifs are frequently involved in signalling events. Indeed, for FAK, P878A/P881A mutation were associated to decrease expression levels of markers for epithelial-mesenchymal transition in vivo in a mouse model of human breast cancer which correlate with an inhibition of the FAK/Endophilin A2 interaction [156]. Thus, targeting such interactions with small molecules or peptide-based sequences may be of potential interest for clinical applications. PR sequences typically contain a Pro-Xaa-Xaa-Pro motif, which folds into a polyproline helix that bind to SH3, WW, GYF or EVH1 domain. The typical Kd of these domains for their respective targets range over 1 to 500 µM which cannot be considered as high affinity. All these domains recognize several residues within the core motif of PR domains. Often aromatic side chains of the PR-binding domain recognize defined proline residues. Selectivity is therefore achieved via specific interaction with core-flanking epitopes and exposed side chains on the surface of the PR domain called epsilon determinants [157]. The determination of the precise characteristic of these critical interactions using structure-based molecular modelling together with the screening of peptide libraries may help the search of PPI targeting PR domains.

### 3.4. Major Interactions at the FAT Domain

#### 3.4.1. FAK Binding to Paxillin and the Targeting to Focal Adhesions

**Figure 4:**
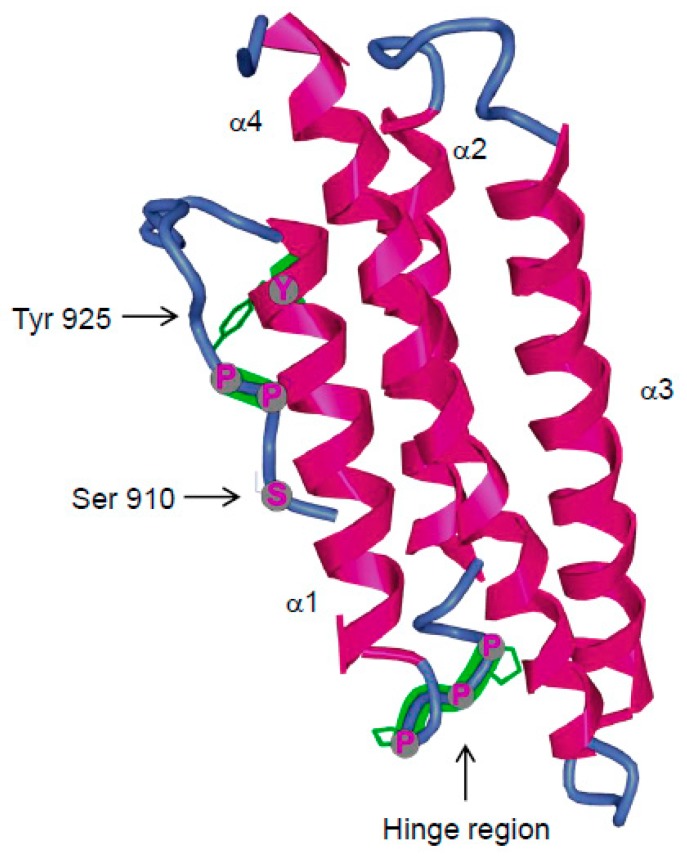
Structure of the FAT domain of FAK from the protein databank (accession code 3S9O). The FAT domain is a four-helix bundle composed of helix α1, α2, α3 and α4. The hydrophobic patches HP1 and HP2 important for paxillin binding are located at the interface of helix α1–α4 and α2–α3. Tyr925 and Ser910 are highlighted. The residues of the hinge domain located between helix α1 and α2 important for FAK dimerization and phosphorylation of Tyr925 are coloured in green. Proteins associating with the FAT domain are shown in the table.

The C-terminal domain of FAK interacts with several FA-associated proteins including Paxillin and Talin [158,159,160], p130^CAS^ [132,161], Grb2 [159,162], the p85α subunit of PI3K [130] and VEGFR-3 [163]. Furthermore, the C-terminal domain is both necessary and sufficient for FAK localization at FAs and thus has been called the FAT domain (Focal Adhesion Targeting). Structurally this domain is a four-helix bundle carrying two hydrophobic patches HP1 and HP2 that mediate interaction with the LD2 and LD4 motifs on Paxillin [164,165,166] (Figure 4). Hence, targeting of FAK to FA has been shown to be mediated primary by binding of FAK to Paxillin although alternative ways, such as binding to Talin, have been described [160]. Classical models postulate that upon Integrin engagement to the ECM, Talin and/or Paxillin are recruited to nascent adhesions via binding to the cytoplasmic tail of Integrin. Indeed, early studies have shown that FAK interact with Talin within the FAT domain and, because *in-vitro* association of Talin with the Integrin β1 cytoplasmic domain has been detected, it was suggested that Talin may target FAK to FAs [160]. However, recently it was clearly demonstrated that FAK acts upstream of Talin and recruits it via FAT binding functions to nascent adhesions, although this mechanism does not take place for Talin recruitment at mature adhesions [167]. Studies using cells from Paxillin knock-out mice or deletion of FAK-Paxillin binding sites revealed an essential role for Paxillin in targeting FAK to FAs although it should be noted that some residual FAK localization at FAs is frequently observed [168,169]. Moreover, overexpression of Hic-5 a Paxillin homologue that also localized to FAs, sequestered FAK from Paxillin and thus reduced phosphorylation of Paxillin and FAK. Therefore, Hic-5-mediated inhibition of cell spreading can be attributed to a competition with paxillin for FAK and subsequent prevention of downstream FAK signalling [170]. Alternative routes for FAK localization at FAs may involve the FERM domain of FAK which enables either FAK interaction with the Arp2/3 complex or FAK targeting to membranes and further binding to PtdIns (4,5) P_2_ at sites of Integrin activation thereby connecting FAK to nascent adhesions [59,70]. Other proteins containing LD motifs have also been shown to bind to the FAT domain of FAK. This includes the tumour suppressor DLT1 (deleted in liver cancer 1) which encodes a Rho-GAP catalytic domain that negatively regulates Rho-GTPases [171] and the delayed rectifier Kv2.1 potassium channel which upon FAK interaction promotes polarized cell morphology and enhanced motility [172]. Interaction between the FAT domain and the FERM domain of FAK has also recently been shown to be involved in FAK dimerization and activation. Surprisingly, the FAT-FERM interactions seem further stabilized by paxillin binding to FAT [65]. Moreover, beside the targeting of FAK to FAs and its subsequent implication for adhesion and migration processes, the FAK-Paxillin interaction is also involved in the regulation of mitotic spindle orientation, thus controlling tissue morphogenesis [173].

#### 3.4.2. FAK Phosphorylation at Tyr925

One important motif of the FAT domain is the Tyr925 phosphorylation site, which has been identified as a Src-dependent process because its phosphorylation is significantly reduced in cells expressing a kinase-defective mutant of Src [180]. Upon phosphorylation it creates a binding site for the SH2 domain of the adaptor protein Grb2 with further activation of the MAP kinase pathway [159]. This pathway has been implicated in the control of VEGF expression in breast carcinoma cells and, when inhibited by selective mutation of FAK at Tyr925, leads to reduced tumour growth in mouse associated with impaired neo-vascularisation [181]. Via the FAK-Grb2 association, Grb2 was also proposed to link FAK to PTPα promoting PTPα phosphorylation to control downstream Integrin signalling [182]. In addition, phosphorylation of this site has been associated with activation of the p130CAS/Dock180/Rac1 signalling pathway-mediated cell protrusion [93] which, at least in Src-transformed fibroblasts, leads to increased matrix metalloproteinase expression and activity and thus to mechanisms underlying cell invasion [94]. Nevertheless, direct association of p130CAS to FAK has been shown to be preferentially mediated by the p130CAS SH3 domain binding to the PR1 FAK domain as previously discussed. Thus, although Dock180 co-immunoprecipitates with FAK, it is unlikely that Dock180 directly interacts with FAK but instead, Dock180 and p130CAS are rather part of a signalling complex which includes FAK and Src. Therefore, the precise molecular interactions leading to activation of this pathway upon phosphorylation of FAK Tyr925 remains to be elucidated as is the search for other SH2-countaining proteins that could interact with this phosphorylated site.

Cell motility also requires the coordinated turnover of nascent adhesions at the leading edge and the disassembly of FA at the rear, a process dependent upon FAK phosphorylation at Tyr925. Because phosphorylation of Tyr925 and subsequent binding of SH2-containing protein require rearrangement of the FAT structure, it has been suggested that this process would lead to alteration of FAK-Paxillin interactions thus ultimately leading to FAK removal from FA [174]. However, although phosphorylation of FAK at Tyr925 increases FA dynamic, it also contributes to increasing the time residency of FAK at FAs and therefore may not be the molecular determinant necessary for FAK removal from FA [93]. On the other hand, rearrangement of the FAT domain involving opening of helix1 from the four-helix bundle may be a conformational change triggering the removal of FAK from FAs. Indeed, this structural rearrangement would alter the conformation of the hydrophobic patch HP2 thus reducing FAK-Paxillin interaction and also allow easy access to Tyr925 for kinase-mediated phosphorylation and subsequent binding of SH2-containing proteins. Indeed, selective deletion of Q943/A945 in the FAT domain of FAK, aimed at inducing increased strain generated by the prolines in the H1-H2 hinge region leads to an increased probability of helix 1 opening as observed by decrease FAK-Paxillin interaction and increase phosphorylation at Tyr925 [175]. Nevertheless, the nature of the signal leading to opening of the FAT domain and subsequent phosphorylation at Tyr925 remains to be determined.

#### 3.4.3. FAK Phosphorylation at Ser910

Early studies have shown that agonists of G protein-coupled receptors, including Bombesin and lysophosphatidic acid stimulate FAK phosphorylation at Ser910, through an ERK-dependent pathway. Moreover, growth factors that stimulate the ERK pathway in 3T3 fibroblast, like FGF, phosphorylate FAK at Ser910 whereas others like Insulin which is unable to simulate the ERK pathway does not promote FAK phosphorylation at Ser910 further indicating the requirement of ERK for phosphorylation at this site [176,183]. Interestingly, phosphorylation of Ser910 in response to Bombesin was increased in a tense FAK mutant with deletion at Q943/A945 that induces destabilization of the FAT domain suggesting that FAT rearrangement also increases Ser910 accessibility to Ser/Thr kinases [175]. Ser910 is located just before a proline rich domain containing P911/P912/P913. Typically, proline-rich domains that binds to SH3 or WW containing proteins are composed of a Pro-X-X-Pro motif but a subset of WW domain bind specifically to pSer/Thr-Pro motifs. The enzymes responsible for the phosphorylation of Ser/Thr-Pro motifs are Pro-directed protein kinases, a large family of kinases which include for example MAPKs, GSK-3 and JNKs. Proline residues exist in both cis and trans isomers catalysed by peptidyl–prolyl cis/trans isomerases. A specific peptidyl–prolyl cis/trans isomerases of the Parvulin family called PIN1 (protein interacting with NIMA1) catalyses the isomerization of Ser/Thr-Pro motifs once phosphorylated. Recently it was shown that the phosphorylation of FAK at Ser910 enables the binding of PIN1thus leading to the recruitment of a protein tyrosine phosphatase PTP-PEST. This in turn leads to the dephosphorylation of FAK at Tyr397 [177]. Therefore, surprisingly in this study the inhibition of FAK via dephosphorylation at Tyr397 is linked in Ras-transformed fibroblast to the ability of Ras to induce cell migration, invasion and metastasis. Considering the overall data indicating a positive role of FAK in tumour progression, the authors suggest that FAK is required for tumour development induced by oncogenic proteins which activate a positive FAK/Src feedback loop whereas in tumour with aberrant Ras which do not rely on Src for their oncogenic development, a MAPK pathway leading to reduce FAK activity is activated that promote invasion and metastasis.

#### 3.4.4. FAK as a Regulator of the Rho-GTPase Family

We described previously interactions between Rho-GTPase family regulators with proline-rich domains of FAK but FAK also interacts via its FAT domain with some of these regulators. First, as discussed before, FAK interacts directly or via formation of a complex that include p130Cas, with Dock180, a GEF for Rac thereby promoting lamellipodia formation. FAK also regulate Rho via interaction with p190RhoGEF that stimulate Rho activity. Although the interaction domains were not precisely mapped, mutation of FAK^L1034S^, which destabilizes the four-helix bundle of the FAT domain, disrupts FAK interaction with p190RhoGEF thus clearly indicating the requirement for an intact FAT domain [178]. Another protein called GIT1, for G-protein-coupled receptor kinase (GRK)-interacting targets which contains at the N terminal a motif functioning as Arf GAP was found to directly associate with FAK via a region closely related to the yeast Spa2 homology domain 1 (SHD-1) [184]. GIT1 is a multifunctional protein involved in focal complex disassembly able to bind Paxillin and the PAK/β-PIX complex (p21-activated kinase/PAK-interacting exchange factor PIX). PAK is an effector for Rac1 and Cdc42 whereas β-PIX is a GEF for Rac. GIT1, like FAK, encompass Paxillin binding motifs that allow direct interaction with the LD4 motif of Paxillin, supporting the notion that GIT1 may regulate FAK/Paxillin interactions and thus focal complex dynamics. Moreover, in NIH3T3 cells, lysophosphatidic acid-induced GIT1-dependent β-PIX binding to FAK thereby involving FAK to both the control of lamelipodia formation via Rac activation and focal complex dynamic via regulation of FAK/Paxillin binding [179]. Nevertheless, the precise role of FAK in this regulation pathway leading to cell polarity and directional migration is even more complex. Indeed, phosphorylation of GIT1 by the FAK/Src complex is required for GIT1 association with FAK at FAs [185]. On the other hand, phosphorylation of GIT2, also called PKL for paxillin kinase linker, by the same FAK/Src complex in response to platelet derived growth factor (PDGF) stimulation, is required for GIT2 association with Paxillin and control of wound-induced cell polarization as well as directional migration [186]. Another regulator of cell polarization and directional migration is Eps8 (Epidermal growth factor receptor kinase substrate 8), initially described as a substrate for EGFR involved in the Ras signalling pathway and endocytosis. In squamous cell carcinoma, Eps8 interacts with FAK at FAs and is required for FAK-dependent polarization and invasion [187]. This interaction require binding of Eps8 to the FAT domain of FAK spanning the region of the amino acids 981–1053, whereas in the absence of FAK, Eps8 interacts with Src to control its trafficking to autophagic structures. These findings demonstrate complex mechanisms by which FAK can function in the regulation of Rac, Rho and Cdc42 to control cellular polarization, lamellipodia extension and directed migration.

#### 3.4.5. Inhibitors of FAT Interactions

FAK localisation at FA is essential for its regulation by Integrin signalling as demonstrated using replacement of the FAT sequence of FAK [188]. Historically, the first inhibitor of FAK described was the endogenous and autonomously expressed inhibitor FRNK (Focal adhesion kinase-Related Non Kinase) [189]. Indeed, early experiments using FRNK, indicated that many aspects of FAK function, especially adhesion and migration, involve FAK targeting to FAs [190,191]. Therefore, one way to inhibit FAK function in cancer cells would be to interfere with this targeting at the level of FAT. However, it was recently shown that FRNK inhibits growth and survival by direct binding to FAK in an inhibitory complex. This inhibition could be relieved by phosphorylation of FRNK at S217 which is the analogue of Ser910 for FAK [192]. Nevertheless, recently it was observed that altering FAK/Paxillin interactions via specific mutations in the FAT domain reduce adhesion and migration processes in non-cancer cells while inhibiting invasion in Src-transformed mouse fibroblasts [193]. Interestingly, the reduced capability of adhesion, migration and invasion observed in cells lacking FAK targeting to FA was greater to complete FAK knock-out demonstrating a gain of function which might be due to an unexpected ability of the mutated form of FAK to sequester key binding partners outside FAs. Thus, development of molecules targeting FAK interaction with protein involved in the localisation of FAK at FAs, may be a valuable strategy for the search of novel FAK inhibitors. A successful illustration of such a strategy came from William Cance’s group who reported that the FAT domain of FAK is also able to bind to VEGFR-3 in different cancer cell types. They further mapped this interaction to a region containing amino acid 853-1052 of FAT. Inhibiting this interaction with a peptide derived from the VEGFR-3 sequence coupled to a TAT cellular penetration motif decrease cell proliferation and induces apoptosis in breast cancer cells [163]. Going a step forward by using the crystal structure of FAT for molecular docking of small compounds targeting the site of interaction with VEGFR-3, they found that chloropyramine hydrochloride, a small molecule previously reported as a H1 histamine receptor antagonist, were able to alter FAK/VEGFR-3 interaction. This led to inhibition of cell proliferation in-vitro and reduction of tumour growth in-vivo in mice bearing breast cancer cell xenografts [194]. Once the lead compound had been validated, they further designed and synthesised analogues of this compound to be tested for binding to FAT and anti-cancer activities. From this second screening, analogue 29 was found to show enhanced specificity and selectivity for FAK-VEGFR-3 interaction and display in-vitro anti-tumour effect in a variety of cancer cell lines including pancreatic and breast cancer cell, glioma and melanoma [195].

## 4. Conclusions

Successful examples have now proven the efficiency of PPI inhibitors as some of these already reached clinical trials. FAK is at the crossroads of integrin and growth factors signalling and, as FAK is overexpressed in many human cancers, FAK is frequently associated with the promotion of oncogenic signals and the inhibition of tumour suppressive pathways. This makes FAK a clearly interesting cancer target. The development of PPI inhibitors targeting FAK is thus quite promising as FAK, via several specific subdomains and numerous sites of phosphorylation, is at the heart of an important interactome. Several small molecules targeting FAK kinase function are currently being developed with some success in clinical trials. Especially, targeting FAK in combination with therapies against other signalling pathways may be a promising approach for the treatment of drug-resistant cancers. Therefore, with the mapping of many FAK interactions at the molecular level, the search for inhibitors of FAK scaffolding function may lead to new promising therapeutics.

## Figures and Tables

**Table:** 

**Interactions**	**References**
Integrin, PIP2, Tetraspanin	[27,37,39,41,42,50,51]
PDGFR, EGFR, IGFR, RET, c-MET	[31,44,45,46,47]
Cadherin, Ezrin, Dynamin	[48,49,52]
Arp2/3, N-WASP, RACK1, ETK	[53,54,55,56,57]
PIAS1, RIP, p53, Mdm2, Nanog	[58,59,60,61,62]

**Table:** 

**Interactions**	**References**
Src, PI3K, PLCγ, PTEN	[86,95,96,97,98,99,100]
Grb7, SOCS3, p120RasGAP	[71,101,102,103]
Catenin, p190RhoGAP	[48,71]
PDZ-RhoGEF, FIP200	[104,105]

**Table:** 

**Interactions**	**References**
Paxillin, Talin, p130Cas	[129,155,157,158,160,162]
Grb2, PI3K, VEGFR3, DLT1, Kv2.1	[127,156,159,160,168,169]
PTPα, ERK, PIN1, p190RhoGEF	[93,173,174,175,176]
GIT1, Eps8	[177,178,179]

**Table 1:** Proteins associating with the PR domains are shown.

Interactions at PR Domain	References
Trio, Src, PI3K, p130CAS, Calpain	[125,126,127,132,133]
Cortactin, MT1-MMP, Ambra, PSGAP	[134,137,138,139,140]
Graf, ASAP1, IQGAP1, CDK5, Dynein	[141,142,143,144,145,146]
